# Tackling gender-based violence in public health workplaces in India: A call for systemic change

**DOI:** 10.1371/journal.pgph.0004327

**Published:** 2025-03-24

**Authors:** Priya Das, Manisha Gupte, Monalisha Sahu, Shubha Nagesh

**Affiliations:** 1 Principal Consultant and Gender Lead, Oxford Policy Management, New Delhi, India; 2 Co-Convener, Mahila Sarvangeen Utkarsh Mandal (MASUM), Pune, Maharashtra, India; 3 Associate Professor & Head, Department of Occupational Health, All India Institute of Hygiene and Public Health, Kolkata, India; 4 Advocacy Advisor- Global Health, Women in Global Health, Dehradun, Uttarakhand, India; PLOS: Public Library of Science, UNITED STATES OF AMERICA

## Introduction

In India’s public health sector, gender-based violence (GBV) plagues the healthcare system. The tragic Kolkata [1] case, where a female healthcare worker was subjected to severe violence while on duty, is part of a larger pattern that highlights the urgent need for better protection and greater support for women in the health sector. India improved its ranking on the Gender Inequality Index (GII) from 122nd in 2021 to 108th in 2022, but the gender-gap continues. According to the National Family Health Survey-5 (2019-20), nearly one-third of women in India have experienced physical or sexual violence, even with self-reporting.

Child marriage persists at a rate of 23%, and almost 49% of women aged 15 to 49 lack decision-making power over how to spend their own money [2]. In this context, cases like Kolkata become symptoms of the gender-based structural inequities and patriarchal norms pervasive in our social fabric, policies and institutional structures. Inadequate implementation of policies and provisions compromises safety Systemic and structural inequities are evident in tokenistic and inadequate implementation of policies and provisions. As a recent study in India reconfirms, safety and security in healthcare settings is worsening with a significant lack in security infrastructure [3]. Healthcare settings often lack gender-responsive infrastructure, such as dedicated duty rooms, toilets, and well-lit spaces.

The implementation of the Prevention of Sexual Harassment (POSH) Act, 2013, enacted by the Government of India with the aim of ensuring a safe and secure working environment for women, free from sexual harassment, remains severely deficient in healthcare settings [4]. Workplace violence remains largely underestimated due to underreporting and informal complaints. The low status and economic vulnerability of female healthcare workers silence many.

Community health workers, the majority of whom are women, face tremendous challenges, particularly in rural areas [5]. They work in unsafe conditions, lack protective policies, and are vulnerable to harassment during field visits, putting their safety and well-being at constant risk.

### A continuum of micro-aggression to gender-based violence

Violent sexual assault is the extreme end of a spectrum of gender-based workplace microaggressions. Micro-aggressions extend beyond physical or sexual assault and include emotional, psychological, and economic harm [6]. Small actions, over time, create hostile workplaces that hinder women’s career growth, limit leadership opportunities, and leave them invisible and undervalued [7].

Within health care, microaggressions can include discrimination, bullying, harassment, and both verbal and non-verbal hostility towards women [8]. Additionally, research has shown that gender bias, sexism and misogyny in mentorship and training environments can undermine women’s professional growth and lead to inequities in patient care. This can manifest in subtle ways, such as the undervaluing of women’s input, lack of opportunities for advancement, and the perpetuation of gender stereotypes [9].

Microaggressions are often dismissed as harmless fuel, a culture of silence and marginalization in healthcare [10]. They become the firmament for perpetuating all forms of sexual exploitation, harassment, abuse and violence. To tackle gender-based violence in public health, we must address its roots, not just the aftermath.

### Gender norms, occupational segregation, and the blurring of public and private spheres

Globally, 70% of healthcare workers are women, yet leadership roles are predominantly held by men, perpetuating systemic inequities [11]. The gender-based occupational segregation within health systems is underpinned by ‘gender essentialism’ and ‘male primacy’, reflecting gender-based perceptions and stereotypes about women’s and men’s traditional roles in society at all levels – the household, community, organisational, and institutional [12].

The concentration of men in senior positions of power within healthcare creates a toxic hierarchy.

where sexual harassment and violence thrive. Although data on GBV against healthcare workers in.

India are unavailable, global studies reveal high prevalence of workplace violence and sexual exploitation, abuse, and harassment (SEAH) against women healthcare workers due to the prevailing gender-imbalance and male-dominated hierarchies within health systems [13,14]. In India, class and caste difference between doctors and mostly female nurses and community level front line workers exacerbates gendered consequences.

The false divide between public and private spheres traps women health workers in a cycle of discrimination. They enter private spaces for work yet face societal expectations to balance caregiving with professional duties. Public and private patriarchies work hand in hand to disenfranchise women in both spheres, pushing women into lower-paying, lower-status roles with heavier workloads, while men take on high-status, decision-making positions. This reinforces the gender divide in the workplace, limiting women’s career growth and opportunities.

### Toward inclusive leadership and effective policies

Institutions must take responsibility for creating safe and inclusive workplace cultures. Legal frameworks like the POSH Act are crucial, but they are not enough. We require more gender equitable health systems, beginning with transparent grievance redressal mechanisms, accountability structures, and proactive training on gender inclusivity and diversity at all levels of clinical and managerial cadres. We need stronger policies to tackle both overt and subtle gender based discrimination in healthcare— from occupational segregation to inadequate security.

Gender-responsive leadership is key to transformation. We urgently need more women in leadership roles to drive change and challenge inequalities in healthcare [15] Leadership is not just about filling a position of power—it is about creating a culture that empowers everyone to succeed, regardless of their gender. Healthcare leaders, including and especially men in decision-making roles, must not only champion gender equity but also be held accountable for progress—or the lack thereof.

## Conclusion: a call to action for systemic change

The conversation about gender-based violence in India’s public health sector is long overdue. Effective change requires dismantling the systemic barriers that perpetuate gender-based violence and discrimination. We must establish benchmarks where leadership reflects the gender of the workforce, with targeted protections for the most vulnerable. Public reporting of gender-based violence data and accountability mechanisms is essential for transparency and driving meaningful change.

The future of India’s public health sector hinges on the safety, dignity, and empowerment of its

workforce. It’s time to build a system where all women—whether in leadership or on the front

lines—can thrive free from fear, violence, and bias.
